# What Happens to Children’s Mental Health and Peer Relationships During Periods of Restricted and Unrestricted Social Interactions? Results From the Co-SPACE Study in Primary School-Aged Children

**DOI:** 10.1016/j.jaacop.2023.05.003

**Published:** 2023-06-06

**Authors:** Carolina Guzman Holst, Sinziana I. Oncioiu, Cathy Creswell, Lucy Bowes

**Affiliations:** University of Oxford, Oxford, United Kingdom

**Keywords:** children, mental health, peer-aggression, peer-victimization, pandemic

## Abstract

**Objective:**

Children’s experiences of peer victimization and peer aggression are strongly linked to their mental health. However, we do not know how this relationship is influenced by periods of restricted and unrestricted social interactions. In this study, we investigated the following: (1) the bidirectional association between children’s peer problems and mental health; (2) individual differences in the joint development of peer victimization, peer aggression, and mental health; and (3) factors associated with joint trajectories over 13 months during the COVID-19 pandemic in the United Kingdom.

**Method:**

Our sample consisted of 2,160 children 4 to 10 years of age for whom parents or carers/caregivers completed a baseline and at least 1 follow-up online survey between March 2020 and May 2021 as part of the COVID-19: Supporting Parents, Adolescents and Children during Epidemics (Co-SPACE) study. We used generalized linear models to examine bidirectional associations, group-based trajectory modeling to map joint trajectories, and multinomial logistic regressions to identify factors associated with trajectories.

**Results:**

Experiencing mental health difficulties during school closures increased the risk of experiencing peer victimization, but not peer aggression on return to school. Experiencing any peer problems during school closures did not predict subsequent mental health difficulties. The majority of children followed joint trajectories of low exposure to peer problems and mental health; however 16% to 17% of our sample followed joint trajectories of high exposure to peer problems and mental health. Low family income, family conflict, parental distress, special educational needs, and lack of friendships were associated with high exposure mental health and peer problem trajectories.

**Conclusion:**

Increasing children’s mental health support during periods of isolation may not only reduce concurrent and future mental health problems but may also prevent subsequent peer problems for both vulnerable and non-vulnerable children.

**Study preregistration information:**

Co-SPACE (Covid-19: Supporting Parents, Adolescents and Children during Epidemics); https://osf.io/.

Peer relationships have an important function in children’s social and emotional well-being. Although close friendships can enhance resilience and improve mental health in young people, negative peer experiences such as peer victimization and peer aggression (hereafter collectively referred to as peer problems) can have detrimental impacts on mental health.^1^^,^^2^ Indeed, evidence from population-based longitudinal studies suggests that there is a strong reciprocal relationship between peer problems and mental health problems, including depression, anxiety, conduct problems, and hyperactivity/inattention problems, meaning that victims of bullying and perpetrators of bullying are more likely to develop mental health difficulties, whereas having pre-existing mental health difficulties puts young people at risk for being bullied or bullying others.^3^, ^4^, ^5^, ^6^, ^7^ However, studies have also shown that when bullying victimization and perpetration are targeted through traditional experimental methods such as anti-bullying interventions, bullying moderately decreases on average by 15% to 16% and perpetration by 19% to 20%,^8^ but internalizing mental health outcomes such as depression and anxiety do not substantially improve.^9^ This might suggest that other factors such as relationships with peers and family, parental psychopathology, or an individual’s pre-existing mental health might be influencing this relationship, and that perhaps other, non-traditional methods may provide novel insights into this association. For instance, a natural experiment such as the COVID-19 pandemic, in which social interactions were periodically restricted and then unrestricted, may allow us to understand new aspects of this association and potentially open up new avenues for future research. In other words, we can leverage the disruption caused by the pandemic to examine what happens to children’s mental health when we place a temporary restriction (ie, school closures) that reduces peer victimization or aggression, and what happens when this restriction is lifted.

In the United Kingdom (with some differences across England, Wales, Scotland, and Northern Ireland), all primary schools were closed for in-person teaching from mid-March to June 2020 for the first lockdown, and from mid-December 2020 to early March 2021 for the second/third lockdown, except for children of key workers and children considered to be vulnerable. A timeline of full coronavirus measures from March 2020 to December 2021 can be found at The Institute for Government.^10^

There is no question that children experienced unprecedented disruptions to their social experiences as a result of the COVID-19 pandemic. Repeated school closures, online learning, social distancing guidelines, and transitioning back into in-person teaching at schools led to significant changes in key areas of daily life, with many people raising concerns about the detrimental impact that this could have on children’s mental health and social experiences.^11^ Indeed, some studies found that limited social experiences, including school closures, had a negative impact on children’s mental health.^12^^,^^13^ Another study looking at trajectories of young people’s mental health during the pandemic found that not having a quality and supportive friendship was associated with trajectory groups characterized by high levels of mental health difficulties.^14^

Yet, the impact of these enforced school transitions on peer relationships and their association with mental health during the pandemic is less clear. Although we expected rates of peer problems to decrease drastically because opportunities for face-to-face social interactions were limited, we do not know the extent to which children’s exposure to online interactions or social media use, and potentially cyberbullying, increased to compensate for restrictions. Nevertheless, school closures combined with other measures limiting social experiences also had the potential to decrease social support, to increase children’s emotional and behavioral difficulties, and to lead to a sharp increase in face-to-face peer problems on return to school. Only 1 study to date has examined changes in specific peer problems including peer victimization and peer aggression before and during the pandemic.^15^ In this study, more than 6,000 school-aged children were randomized at the school level to retrospectively report on rates of bullying before the pandemic or during the pandemic. They found that 66% of primary school students reported being bullied before the pandemic compared to 48% during the pandemic, and 14% reported bullying others before the pandemic compared to 7% during the pandemic.^15^ Although this study suggests that bullying victimization and perpetration rates were higher before lockdown than during periods of school closures, there is no study to date examining the joint development of peer problems and mental health throughout the pandemic, when schools closed and re-opened several times.

In addition to studying the relationship between peer problems and mental health and their joint development over time, it is important to consider factors that may be associated with different co-development profiles. Indeed, younger children with pre-existing mental health problems, special educational needs, or neurodevelopmental disorders might be more susceptible to both peer problems and mental health problems^16^^,^^17^ and require additional support transitioning between school closures and re-openings. Furthermore, several other risk factors might also play a role in children’s experiences of peer problems when they return to school and afterward. This includes risk factors specific for peer victimization, such as internalizing symptoms or limited social support,^18^^,^^19^and risk factors for peer aggression including externalizing symptoms and exposure to family conflict, including sibling bullying.^20^^,^^21^ Disentangling trajectories and pathways between peer victimization or peer aggression and mental health, as well as potential risk and protective factors associated with different trajectories, can help us determine which children are more vulnerable and might benefit from additional support.

For the present study, we conducted a preliminary exploratory analysis to determine (1) whether children’s peer problems during school closures predicted subsequent mental health when schools re-opened, and (2) whether children’s mental health during school closures predicted subsequent peer problems, accounting for pre-school closure levels in both pathways. To further examine this association considering individual differences, our aims were as follows: (1) to identify the joint trajectories of (a) peer victimization and mental health and (b) peer aggression and mental health, during the first 13 months of the pandemic, using parent-reported emotional, conduct and attentional/hyperactivity difficulties subscales from the Strengths and Difficulties Questionnaire (SDQ); and (2) to examine whether several known individual-, family-, and peer-level factors such as children’s pre-existing mental health problems, special educational needs (SEN) or neurodevelopmental disorders (ND), child demographics, family conflict, parental psychological distress, and friendship quality and support were associated with specific joint trajectories of victimization or perpetration and mental health.

## Method

### Design

The COVID-19: Supporting Parents, Adolescents and Children during Epidemics (Co-SPACE) study is an online longitudinal study comprising a convenience sample of UK parents and carers/caregivers (hereafter known as parents) of children and young people between 4 and 16 years of age, who were invited to take part in monthly online surveys from late March 2020 to May 2021. The research protocol is available via the Open Science Framework (OSF; http://osf.io/8zx2y/), where more details on the eligibility, recruitment, procedure, and participants can be found. The pre-registered protocol of the current study can be found at: https://osf.io/jtg3h. Deviations from the protocol can be found in Supplement 1, available online. From December 2020, participants had an opportunity to enter a prize draw every month to win one £50 voucher. Ethical approval for the study was provided by the University of Oxford Medical Sciences Division Ethics Committee (reference R69060). Parental consent was obtained for participation in the study and for this report to be published.

### Participants

The current study consisted of a subsample of parents (n = 2,160) who completed a baseline survey for their child (4-10 years of age) between March 30, 2020, and April 29, 2020, and then completed at least 1 of 13 follow-up surveys between May 1, 2020, and May 31, 2021. Only those who completed all items for the emotional, conduct, and hyperactivity/inattention subscales of the SDQ and single items for peer victimization and peer aggression at baseline were included in the analysis. Participants with missing data on predictor variables were included in the analysis. Table 1 shows demographic and baseline characteristics for our sample.

**Table 1:** Sample Demographics and Baseline Characteristics

Characteristic	Frequency (%)	Range (max score)^a^
Child age, y, mean (SD)	7.12 (1.89)	4-10 (10)
Child sex		
Female	1,049 (48.6)	
Male	1,104 (51.1)	
Prefer not to say or missing	7 (0.3)	
Child ethnicity		
White (British, Irish or other)	1,979 (91.6)	
Other ethnic backgrounds	168 (7.8)	
Prefer not to say or missing	13 (0.6)	
Pre-existing child mental health problems		
No mental health problems	2,103 (97.4)	
Mental health problems	57 (2.6)	
Child SEN/ND		
No SEN/ND	1,856 (85.9)	
SEN/ND	304 (14.1)	
Parental psychological distress (SD)	5.35 (4.46)	0-25 (27)
Prefer not to say or missing	3 (0.1)	
Family income		
< £16,000/y (<£310/wk)^b^	95 (4.4)	
> £16,000/y (>£310/wk)^b^	1,938 (89.7)	
£16,000-£29,000/y	218 (10.1)	
£30,000-£59,000/y	675 (31.3)	
£60,000-£89,000/y	524 (24.3)	
£90,000-£120,000/y	277 (12.8)	
> £120,000/y	244 (11.3)	
Prefer not to say	127 (5.9)	
Friendship quality and support^c^		
Has no good friends	1,139 (52.7)	
Has at least 1 good friend	1,020 (47.2)	
Prefer not to say or missing	1 (0.0)	
Parent−child conflict, mean (SD)	0.84 (0.63)	0-3 (4)
Prefer not to say or missing	7 (0.3)	
Sibling−child conflict, mean (SD)	1.21 (0.68)	0-3 (4)
Prefer not to say, missing, N/A	530 (24.5)	
Family conflict, mean (SD)^d^	2.07 (1.07)	0-6 (8)
Prefer not to say, missing, N/A	578 (24.7)	

Note: max = maximum; N/A = not applicable; SEN/ND = special educational needs or neurodevelopmental disorders.

a Scores include the sample range and possible score maximum score in parentheses for continuous variables.

b Binary measure of family income used for analysis.

c Parents who responded “a bit” to the item asking whether their child had any friend they could turn to for support were coded as 0 and included in the group of “has no good friends,” as this answer does not seem to fully capture a stable quality and supportive friendship. Changing the code for this rating to 1 (“has at least 1 good friend”) does not change any of the results.

d Family conflict variable includes participants with responses to both parent and sibling conflict.

### Measures

Our main outcomes, including peer problems and mental health measures, were assessed over the first 13 months of the pandemic, and other risk and protective factor measures were assessed only at baseline (time 0). Main outcomes using the SDQ were assessed “over the past 6 months” at baseline (in line with SDQ guidance) and “over the past month” at subsequent waves, as follow-ups occurred monthly.

#### Peer Victimization and Peer Aggression

Peer victimization and peer aggression were assessed using the SDQ single-item questions “Picked on or bullied by other children” (peer victimization) and “Often fights with other children or bullies them” (peer aggression), which were both rated using a 3-point Likert scale from 0 (“not at all”) to 2 (“certainly true”). Responses were dichotomized by “not at all,” coded as 0, and “somewhat true” or “certainly true,” coded as 1.

#### Child Mental Health

Parents completed the parent-report version of the Strengths and Difficulties Questionnaire (SDQ),^22^ which is a widely used measure of mental health in children. This study used 3 of the 5 subscales that focus on mental health: emotional symptoms, conduct problems, and hyperactivity/inattention problems. The peer aggression item was excluded from the conduct subscale in the peer aggression model. Each subscale consists of 5 items (4 in the conduct subscale) scored using a 3-point Likert scale ranging from 0 (“not at all”) to 2 (“certainly true”). SDQ scores were coded based on the 4-categorization band for the parent-rated SDQ: low, moderate, high, very high (see www.sdqinfo.org). For more details on coding, see Supplement 1, available online.

#### Child’s Pre-existing Conditions

Parents reported on whether their child had any pre-existing diagnosed mental health disorders (depression, anxiety, or other) and special educational needs or neurodevelopmental disorders (autism spectrum or attention-deficit/hyperactivity disorder). All variables were coded as binary.

#### Child Demographic Information

Parents were asked about their child’s age, sex (male or female), ethnicity (White or non-White), and usual total household income. Income was coded into more than £16,000 per year and less than £16,000 per year, as this reflects an income below 60% of the median income in the United Kingdom.

#### Family Conflict

Parents responded to 2 single-item questions: “My child and I argue a lot” (parent−child conflict), and “My child and their siblings argue a lot” (child−sibling conflict). Each statement is rated on a 4-point Likert scale and summed to derive 1 cumulative continuous variable. Cumulative scores were computed only for participants who responded to both questions.

#### Parent Psychological Distress

Symptoms were assessed at baselinedusing a subset of 9 items^23^ from the Depression Anxiety Stress Scales (DASS).^24^ Each statement is rated on a 4-point severity/frequency scale ranging from 0 (“did not apply to me at all”) to 3 (“applied to me very much, or most of the time”) over the past week. An overall score was obtained by summing the scores for each item, with a total maximum score of 27 and treated as continuous.

#### Friendship Quality and Support

Parents were asked whether their child had “at least 1 friend that they can turn to for support.” This was rated on a 4-point scale: “not at all,” “a bit,” “a lot,” and “completely.” Responses were dichotomized by “not at all” and “a bit,” coded as 0, and “a lot” and “completely,” coded as 1.

### Statistical Analysis

We used descriptive statistics to analyze participant demographic and baseline characteristics using R (v.4.1.2; R Core Team). A time variable was devised based on the month of survey completion, which ranged from 0 for March/April 2020 to 13 for May 2021. If participants had multiple entries per calendar month, then only the first entry was used in the analysis. Missing data was handled using full information maximum likelihood. Attrition was difficult to determine, as respondents were allowed to complete surveys at any follow-up time point (for details, see Supplement 2, Figure S1 and Figure S2, available online).

We conducted an exploratory analysis using generalized linear models with binomial and gaussian distributions to examine the relationship between mental health and individual peer problems during 3 time points (T1, T2, T3), corresponding to times before, during, and after the second wave of school closures. The model used mental health and peer problems during school closures (T2) to predict mental health and peer problems when schools re-opened (T3), while controlling for both before school closures (T1). Only participants with no missing data on these variables were included in this analysis (N = 752) (for full model specification see Supplement 3, Table S1 and Table S2, available online). For our main analysis, we used group-based multi-trajectory modeling (GBTM) to model the trajectories of (1) peer victimization and mental health, and (2) peer aggression and mental health. This modeling approach is based on semi-parametric mixture models and maximum likelihood to identify clusters of children following similar trajectories with multiple exposures over time.^25^ We used a censored normal model for the SDQ subscales and a logit model for peer problems. We did not control for any variables to derive trajectories, as this might affect true latent class formation and classification.^26^ To determine the number of groups, we fitted a series of models between 1 and 8 trajectories with cubic, quadratic, and linear functions. Once the number of groups was established, we improved model fit by examining each individual trajectory function. We determined that a combination of quadratic and linear functions best fit the data for the SDQ subscales and cubic functions for the peer problem items (see Supplement 4, Table S3 and Table S4, available online, for full specifications). Model selection was based on the Bayesian information criterion (BIC), with lower values denoting a better fit.^25^ Model adequacy was based on average posterior probabilities (APP) and odds of correct classification (OCC). Specifically, models with APP values greater than 0.70 and OCC values greater than 5.0 were considered good fits. Other considerations included successful convergence and ease of interpretability. Analyses were conducted using the “traj” procedure in STATA 14^27^; the code syntax is available in Supplement 5, available online. We also examined the associations between baseline factors and trajectory group membership using multinomial logistic regression analysis in R. We excluded the pre-existing mental health variable, as there were not enough cases per group to run the analysis. We conducted sensitivity analysis for the family conflict variable including values for participants with and without siblings to account for any differences (see Table S5, available online). We report odds ratios (ORs) with 95% CIs.

## Results

Our data consisted of 2,160 parental reports of children. Table 1 shows demographic and baseline characteristics for our sample. For details on missing data see Supplement 2, Figure S1 and Figure S2, available online).

### Reciprocal Relationship Between Mental Health and Peer Problems

**Figure 1:**
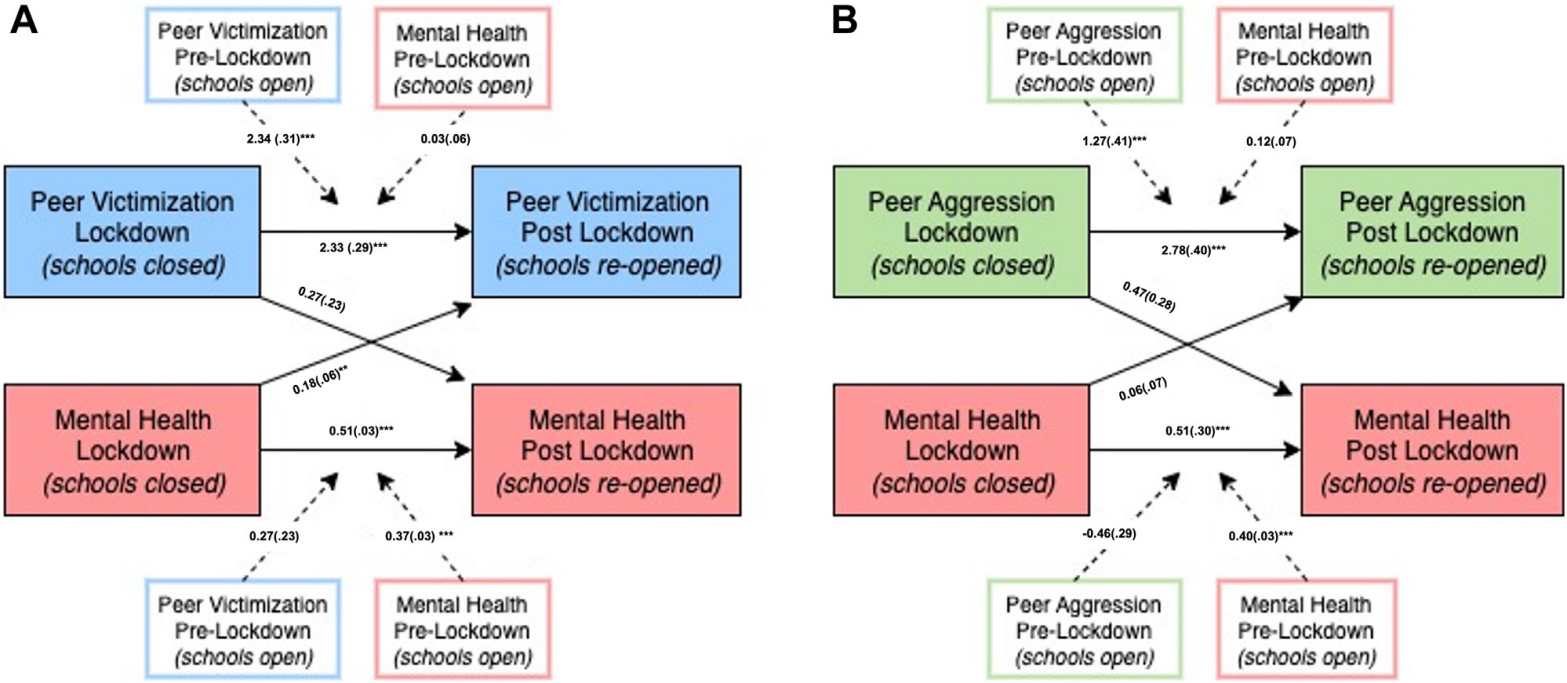
Pathways Between (A) Mental Health and Peer Victimization and (B) Mental Health and Peer Aggression ***Note:****Dotted lines are used to represent the effect of controlled variables (peer victimization or peer aggression and mental health before school closures). Each pathway presents beta (standard error).∗∗*p *< .05; ∗∗∗*p *< .001.*

The peer victimization model is presented in Figure 1B. In this model, mental health measures at T1 and T2 both individually predicted children’s mental health difficulties at T3. Similarly, peer victimization measures at T1 and T2 both individually predicted peer victimization at T3. Mental health at T2 was associated with increased risk of peer victimization at T3, over and above mental health and victimization at T1 (OR = 1.20, 95% CI = 1.07-1.35). Peer victimization at T2 did not predict mental health at T3. The peer aggression model is presented in Figure 1A. In this model, mental health at T1 and T2 individually predicted mental health at T3, and peer aggression at T1 and T2 individually predicted peer aggression at T3. Pathways between peer aggression and mental health were not significant (for all results, see Tables S1 and S2, available online).

### Joint Trajectories of Peer Problems and Mental Health

**Figure 2:**
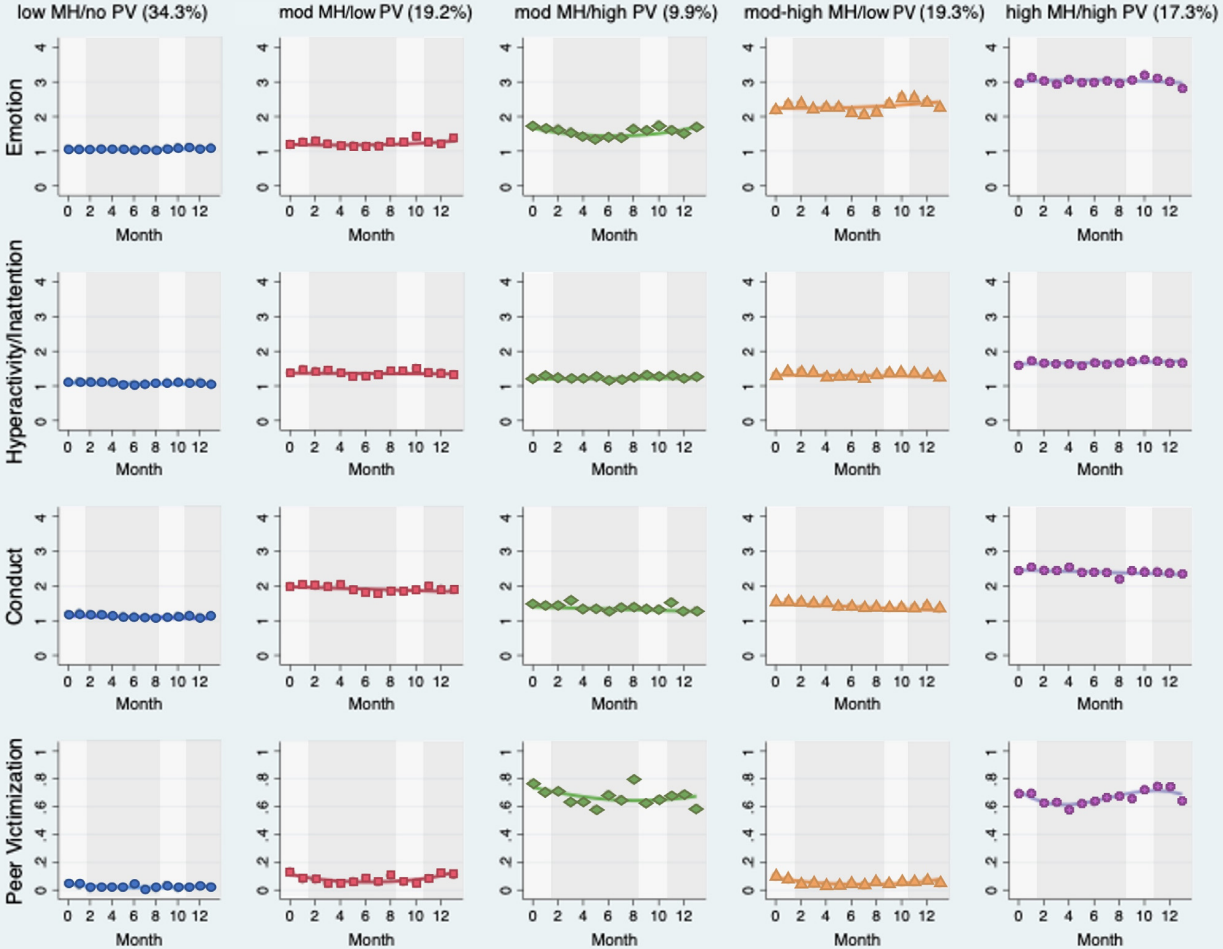
Peer Victimization and Mental Health Trajectories ***Note:****Each color represents a group of children following multiple exposures over time. The x-axis shows time in months, and the y-axis shows each individual exposure. Label and size of each group (given in percentages) can be found at the top of each column. Light gray shading reflects periods of national lockdown, and dark gray shading reflects periods when schools were open (including holiday periods at time points 4 and 5 and time points 7 and 8). MH = mental health; PV = peer victimization.*

Five trajectories were identified for our peer victimization model (Figure 2). In all, 34.3% of children in our sample (n = 759) followed a trajectory characterized by low mental health difficulties and no peer victimization (low stable or low MH and no PV). A total of 19.2% of children (n = 401) followed a trajectory of moderate mental health difficulties and low peer victimization (mod MH and low PV). Another 19.3% of children (n = 416) followed a trajectory of moderate to high mental health difficulties and low peer victimization (mod MH and low PV). A total of 17.3% of children (n = 378) followed a trajectory of high mental health difficulties and high peer victimization (high stable or high MH and high PV), whereas 9.9% of children (n = 211) followed a trajectory of moderate mental health difficulties and high peer victimization (mod MH and high PV). Overall, about 73% of children in our sample followed trajectory groups in which they were exposed to low levels of peer victimization, and almost three-fourths of children in these groups experienced low to moderate mental health difficulties. In comparison, 27% of children followed trajectory groups in which they experienced high peer victimization, and almost two-thirds of children in these groups concurrently experienced high mental health difficulties.

**Figure 3:**
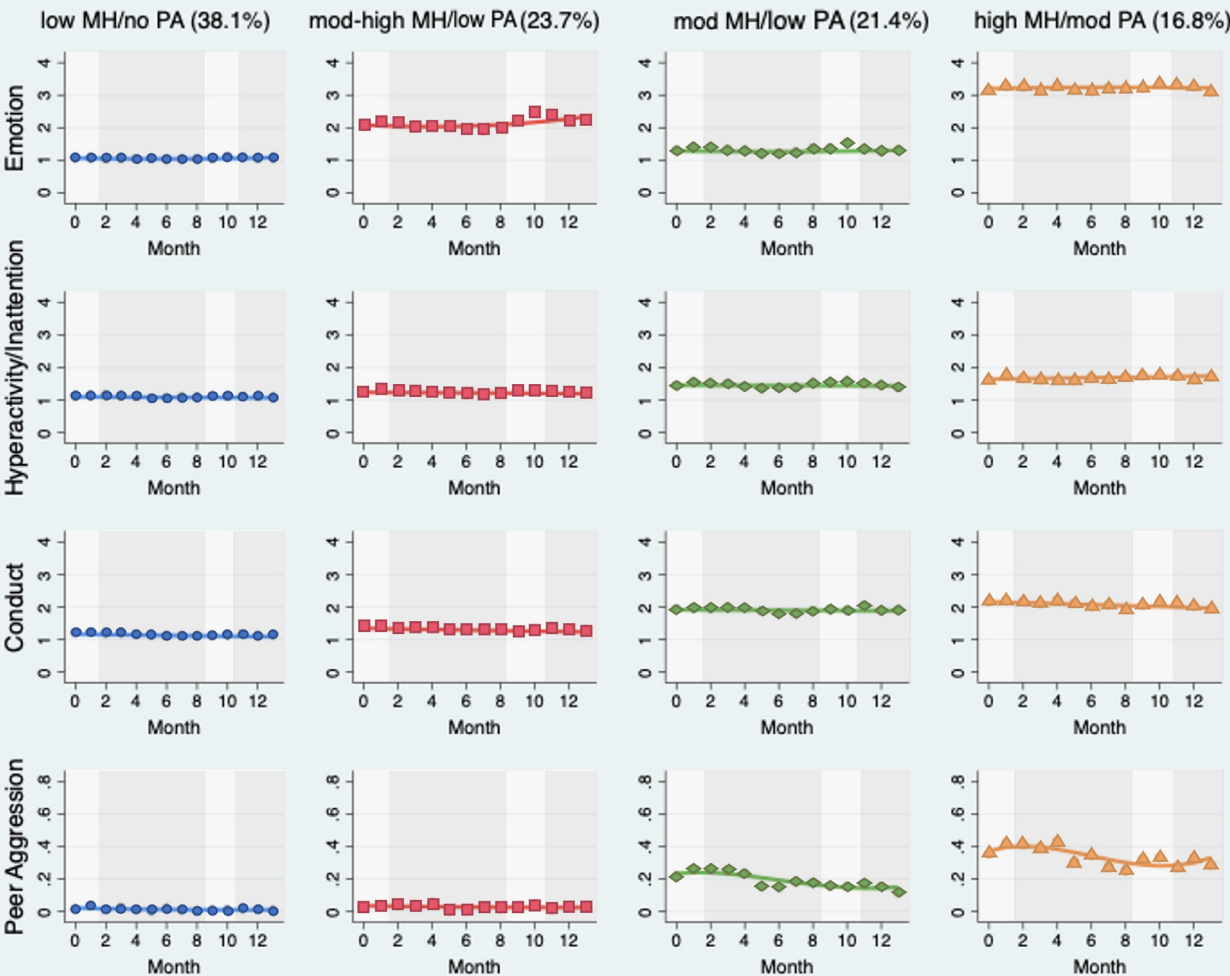
Peer Aggression and Mental Health Trajectories ***Note:****Each color represents a group of children following multiple exposures over time. The x-axis shows time in months and the y-axis shows each individual exposure. Label and size of each group (given in percentages) can be found at the top of each column. Light gray shading reflects periods of national lockdown, and dark gray shading reflects periods when schools were open (including holiday periods at time points 4 and 5 and time points 7 and 8). MH = mental health; PA = peer aggression.*

Four trajectories were identified in our peer aggression model (Figure 3). Of the children, 38.1% (n = 836) followed a trajectory group characterized by low mental health difficulties and no peer aggression (low stable or low MH and no PA); 23.7% of children (n = 511) followed a trajectory group characterized by moderate to high mental health difficulties and low peer aggression (high MH and low PA); and 21.4% of children (n = 455) followed a trajectory characterized by moderate mental health problems and low peer aggression (mod MH and low PA). Finally, 16.8% of children (n = 358) followed a trajectory group characterized by high mental health difficulties and moderate peer aggression (high stable or high MH and mod PA). Overall, 61.8% of children followed trajectories in which they did not experience peer aggression, and almost two-thirds of children in these groups experienced low mental health difficulties. In comparison, 38.2% of children followed trajectories in which they experienced low to moderate levels of peer aggression, and almost half of the children in these groups experienced concurrent high mental health difficulties.

### Factors Associated With Joint Trajectories of Peer Problems and Mental Health

#### Peer Victimization

**Figure 4:**
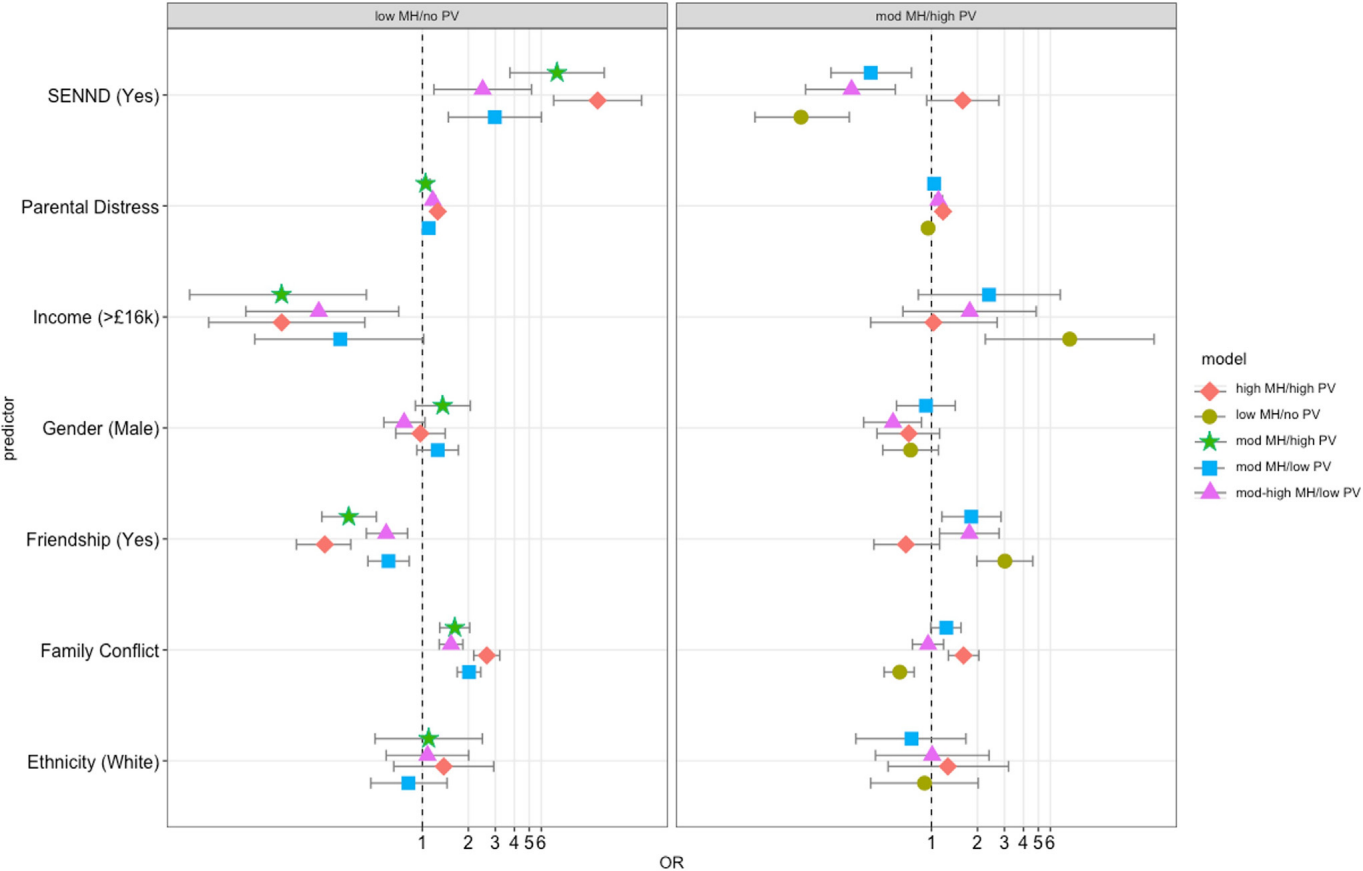
Factors Associated With Trajectory Membership in the Mental Health and Peer Victimization Model, Using High and Low Stable Groups as a Reference ***Note:****This figure shows odds ratios (ORs) with 95% CIs. The reference for friendship is that a child does have at least 1 close and supportive friendship. Family conflict and parental distress are measured continuously, with higher scores corresponding to higher levels. CIs for parental distress are too narrow to be visualized. SENND = special educational needs or neurodevelopmental disorder.* ***∗****Statistically significant OR at* p *< .05.*

Compared to children in a trajectory group characterized by low levels of mental health difficulties and no peer victimization (referred to as the low stable group or low MH/no PV), children in all other groups were significantly more likely to have an SEN/ND and to experience high levels of family conflict, and were less likely to have at least 1 good friend to turn to for support (Figure 4). Children in most groups compared to the low stable group were significantly more likely to have parents reporting higher levels of psychological distress (except for those in the mod MH and high PV group) and more likely to come from low-income households (except for those in the mod MH and low PV group, which were only marginally significant). Trajectory groups did not differ in terms of sex or ethnicity. In addition to our main analysis, we conducted 1 additional exploratory analysis using the trajectory with moderate mental health and high peer victimization (mod MH and high PV) as a reference, to tease out differences that may arise from groups with high victimization and high mental health and high mental health only. Children in the group experiencing mod MH and high PV were less likely to have a supportive friend and more likely to have an SEN/ND compared to those experiencing mod-high MH and low PV. They were also less likely to have a parent reporting high levels of psychological distress and to have experienced lower levels of family conflict compared to the high MH and high PV group (for all regression results, see Supplement 6, Tables S6 to S9, available online).

#### Peer Aggression

**Figure 5:**
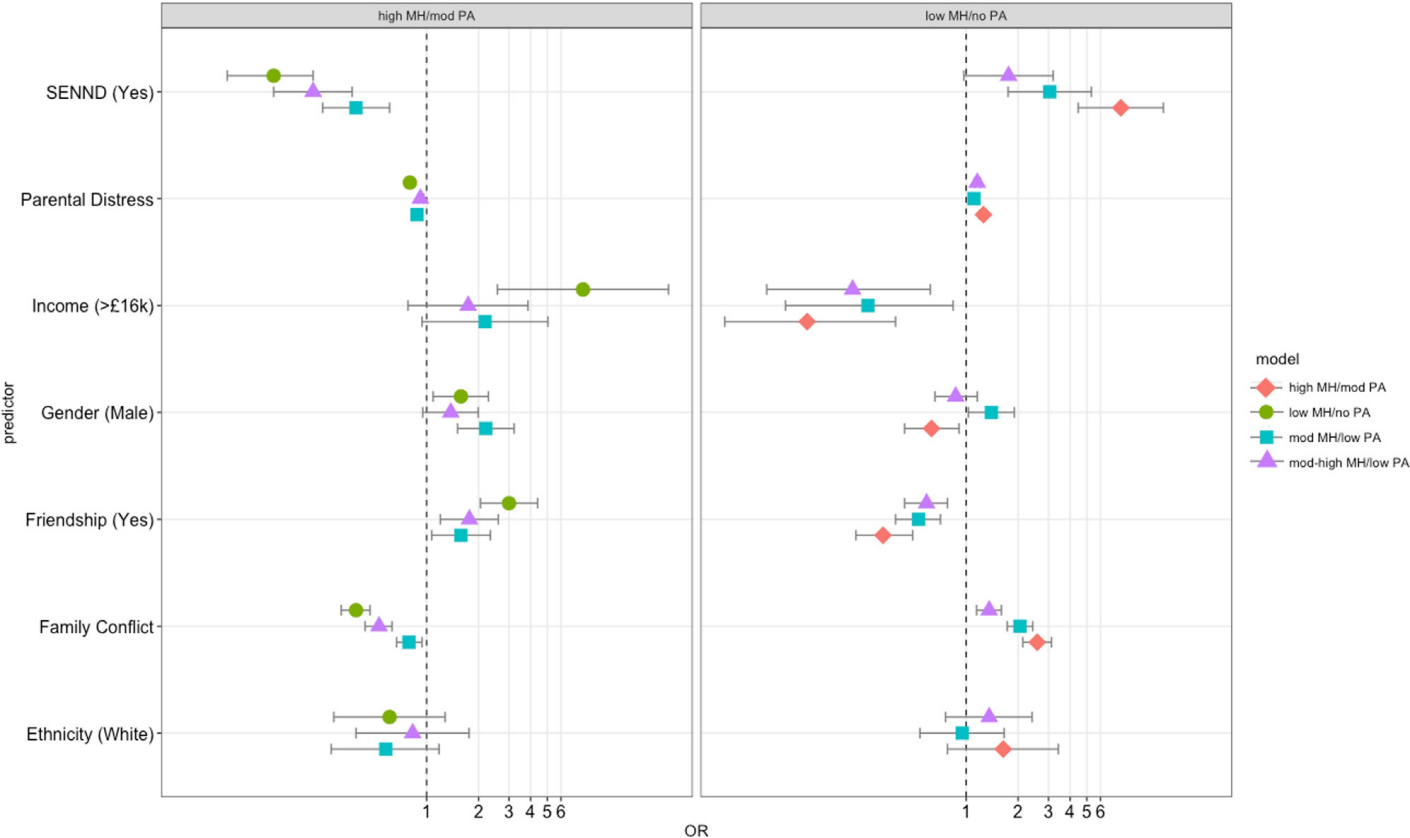
Factors Associated With Trajectory Membership in the Mental Health and Peer Aggression Model, Using High and Low Stable Groups as a Reference ***Note:****This figure shows odds ratios (ORs) with 95% CIs. The reference for friendship is that a child does have at least 1 close and supportive friendship. Family conflict and parental distress are measured continuously, with higher scores corresponding to higher levels. CIs for parental distress are too narrow to be visualized. SENND = special educational needs or neurodevelopmental disorder.* ***∗****Statistically significant OR at* p *< .05.*

Compared to children in a trajectory group experiencing low levels of mental health difficulties and no peer aggression (low stable group or low MH/no PA), children in all other groups were more likely to have parents reporting higher levels of psychological distress and to experience higher levels of family conflict, and were less likely come from a higher income family or to have at least 1 good friend to turn to for support (Figure 5). Regardless of mental health, children in trajectory groups experiencing low and moderate peer aggression (but not children experiencing no peer aggression) were more likely to have an SEN/ND compared to children in low stable groups. Children in trajectory groups experiencing low peer aggression were more likely to be male, whereas those experiencing moderate peer aggression were more likely to be female compared to low stable groups. Compared to children in trajectory groups experiencing high levels of mental health difficulties and moderate levels of peer aggression (high stable group or high MH and mod PA), children in all other groups were less likely to have an SEN/ND, to have parents reporting higher levels of psychological distress, and to experience higher levels of family conflict, and were more likely to have at least 1 good friend to turn to for support. Children in low stable and mod MH and low PA were more likely to be male than those in high stable groups (for all regression results, see Supplement 6, Tables S10 and S11, available online).

## Discussion

This study explored the relationship between children’s peer problems, including peer victimization and peer aggression, and mental health during the COVID-19 pandemic. We found that experiencing mental health difficulties during school closures predicted peer victimization when schools re-opened (but not the reciprocal path), and we found no significant association for pathways between peer aggression and mental health. We also analyzed the joint development of mental health and peer problems over the first 13 months of the pandemic. Although most children followed trajectories of low peer problems and low/moderate mental health difficulties, over one-fourth of the sample experienced moderate to chronic peer problems and high mental health difficulties. These groups were associated with more individual and family risk factors when compared to the group with low difficulties.

Our results support current theories and research findings showing that children at low risk for mental health problems are less likely to experience peer victimization, whereas those at high risk for mental health problems are more likely to be victimized.^28^ Even in the pandemic context, a previous cross-sectional study showed that children and young people who reported a perceived improvement in mental well-being also reported reduced bullying and less loneliness and exclusion compared to peers who reported no change or perceived deterioration in mental well-being.^29^ We were able to build on these findings by examining pathways at key points in the pandemic while accounting for prior levels of peer problems and mental health. Our results suggest that intervening or increasing support during periods of social restrictions to improve children’s mental health might positively influence not only children’s current and subsequent mental health but also their peer relationships. Although we do not know the exact mechanisms as to why or how this might happen, it is possible that improving mental health might influence emotions and cognitions related to feelings of loneliness or perceptions of isolation, and might encourage children to seek support or to be more receptive to subsequent social experiences.^30^ Indeed, studies have found that children who experience loneliness appear to have increased levels of depression and social anxiety,^31^ which could have a significant impact on their ability to form and maintain friendships when schools open. Digital interventions that enable remote support for child mental health problems or that enable children to apply and develop social and emotional skills could be beneficial in this context.^32^^,^^33^ Furthermore, these findings may have wider implications for children who are socially isolated because of life events, illness, or other circumstances.

As previously mentioned, many factors can influence the relationship between peer problems and mental health. The presence of other unaccounted factors in our model might explain why we did not find an association between being victimized and subsequent mental health. For example, specific pandemic-related factors such as parental stress, financial deprivation, or other factors not measured in this study, such as bereavement, may very well influence and underestimate children’s mental health above and beyond peer problems. Moreover, using average levels of mental health and peer problems might preclude us from understanding individual differences and identifying children that might follow distinct patterns over time. Thus, to examine our main aim, we used group-based trajectory modeling to avoid generalizations about average effects based on the “majority” of children.

In this study, we identified 5 different trajectories for exposure to both peer victimization and mental health (model 1), and 4 different trajectories for exposure to peer aggression and mental health (model 2). In both models, most children followed low stable trajectories, whereas a smaller portion followed high stable trajectories, indicating that children in our sample were able to adapt and to cope, both emotionally and socially, to the different challenges imposed during the first year of the pandemic, including changes in access to social experiences and support. Notably, similar individual trajectories have been reported for peer problems and mental health separately in other contexts.^34^^,^^35^ For both models, most of the variability in our trajectories came from differences in the severity of mental health. Indeed, moderate levels of mental health were driven mainly by conduct problems, whereas high levels of mental health difficulties were driven mainly by emotional symptoms. Other studies examining trajectories of mental health during childhood have found similar trends emerging distinctly for internalizing and externalizing symptoms over a longer period of time.^36^ Ultimately, our findings and those of other studies demonstrate that children exposed to stressful circumstances might benefit from different levels and types of support to deal with emotional and behavioral difficulties, rather than a “one size fits all” approach. Finally, we found 1 trajectory characterized by high levels of peer victimization and moderate mental health difficulties. This trajectory was rather unexpected, as we would assume that experiencing high peer victimization would be associated with high levels rather than moderate levels of mental health.^19^ It is likely that these children, as opposed to those experiencing high peer victimization and high mental health, had several protective factors in place, some of which might have buffered any negative impacts associated with persistent peer victimization.^37^

Our results showed that known risk factors such as family conflict, parental psychological distress, lower income levels, and having an SEN/ND were associated with high levels of peer problems and mental health difficulties, whereas known protective factors such as having at least 1 supportive friend, having higher family income levels, and experiencing less family conflict or parental psychological distress were associated with low stable levels of peer problems and mental health difficulties. These findings are consistent with other studies during the pandemic showing that children with an SEN/ND, low income, and limited parental support were more likely to experience mental health difficulties.^38^^,^^39^ Building on this, our study also found that peer relationships and quality friendships are just as important in protecting children from following high stable mental health and peer problem trajectories. Future research should examine the role of positive peer relationships during periods of social isolation and their impact on subsequent social functioning.

Our results also suggest that family relationships played an important protective role for children who were highly victimized and followed trajectories of moderate rather than high mental health difficulties. This is in line with other studies that have reported on the protective effects of family relationships during the pandemic.^40^^,^^41^ In contrast, friendships provided a different type of support: they were more closely related to differences in peer victimization than mental health. Independent of mental health severity, children in trajectories with low levels of peer victimization were more likely to have at least 1 quality friend at baseline than those experiencing high levels of victimization. Thus, having a quality friendship may play a role in preventing concurrent peer problems and, potentially, improving subsequent mental health. Indeed, 1 longitudinal study found that youth reporting lower levels of in-person and online socialization, greater isolation, and less peer and parent support developed increased internalizing and externalizing difficulties during the pandemic, even after controlling for pre-pandemic symptoms.^12^ Overall, these findings suggest that both family and peers are both important protective factors that can contribute in different capacities to children’s social and emotional well-being. Identifying other factors and their mechanisms should be a focus for future research.

Our findings should be understood in the context of some limitations. First and foremost, the lack of pre-pandemic data meant that we were not able to comment on how peer relationships and mental health may have changed as a result of the pandemic. Second, it is important to highlight that this study population was not a representative sample, as it was a convenience opportunistic sample biased toward middle and high-income families from White British backgrounds. Moreover, coronavirus restrictions were often changing across the United Kingdom at different times during the pandemic. Since our sample was distributed all over the United Kingdom, we did not have enough statistical power to analyze the impact of these restrictions on monthly evaluations, and thus focused mainly on national restrictions. However, because the sample was spread out, it is unlikely that geographic restrictions had a significant impact on our findings. In terms of the measures used in this study, we did not use multiple informants to validate the peer victimization and aggression items. Given the single-item questions and limited descriptions for bullying and peer aggression, we were not able to distinguish between different types of bullying (eg, verbal, emotional, physical, cyber) and whether they varied at different points in the pandemic, or whether parental reports included sibling bullying occurring at home. We were also not able to examine peer victimization and peer aggression together (eg, to capture bully-victims), as this model did not converge. Although parental reports of children’s peer victimization and mental health have shown to be reliable,^42^ we did not control for parental mental health or other factors when deriving trajectories; thus, is it possible that parents’ perceptions of their child’s mental health might be influenced by their own mental health^43^^,^^44^ and that other pandemic and non-pandemic factors influenced trajectories. Similarly, parental reports of quality friendships might not be as reliable as self-reports; however, we believe that being in prolonged contact with their child at home meant that parents were more aware of their child’s quality friendships. Another limitation is that we examined covariates only at baseline and thus do not consider any changes to these factors that might have occurred throughout the study period. In terms of missing data, it is important to acknowledge that there was a substantial amount of missing data, as many parents did not fill out surveys every month. Even if we used well-established methods to deal with our missing data, our models might not fully represent all trajectories. Finally, we generally caution against drawing any causal inferences about the direction of effects in this study because of unmeasured confounding in our models.

Our findings contribute to children’s social and emotional developmental literature by highlighting the importance of considering individual difference in children’s experiences with limited social opportunities, as these can help us make recommendations that reflect the level of support required by individuals. In this study, we found that supportive peer and family relationships can play an important role in protecting children from experiencing persistent negative social and mental health outcomes, and that additional resources are required to support children experiencing high levels of peer problems, even in circumstances where we do not expect these problems to occur.
