# Supplementary material for: What Happens to Children’s Mental Health and Peer Relationships During Periods of Restricted and Unrestricted Social Interactions? Results From the Co-SPACE Study in Primary School-Aged Children
Source: JAACAP Open. 2023 Jun 6;1(2):128–40. doi: 10.1016/j.jaacop.2023.05.003 (PMC11562425; doi:10.1016/j.jaacop.2023.05.003)
Supplement: Supplemental Data [file mmc1.docx]

**Supplement 1: Notes**

1. Note on deviations from protocol
2. Notes on SDQ scoring

**Supplement 2: Missing Data and Attrition**

1. Note on missing data and attrition
2. Figure S1. Frequency of surveys with all SDQ items completed by a parent across all time points
3. Figure S2. Non-cumulative missing data patterns across SDQ subscales for each time-point

**Supplement 3: GLM Model Specifications**

1. Generalized Linear Models Specifications
2. Table S1. Generalized linear regression results for all pathways between peer victimization and mental health
3. Table S2. Generalized linear regression results for all pathways between peer aggression and mental health

**Supplement 4: GBTM Model Specifications**

1. Table S3. Model selection results
2. Table S4. Model adequacy results

**Supplement 5: Code Syntax**

1. Syntax STATA Group Based Trajectory Modelling

**Supplement 6: Multinomial Regression Analysis and Sensitivity Analysis**

1. Table S5. Sensitivity analysis for family conflict variable
2. Table S6. MNRA peer victimization – Low Stable Reference
3. Table S7. MNRA peer victimization – High Stable Reference
4. Table S8. MNRA peer victimization – Mod MH/ High PV
5. Table S9. MNRA peer victimization – Mod-high MH/Low PV
6. Table S10. MNRA peer aggression – Low Stable Reference
7. Table S11. MNRA peer aggression – High Stable Reference

**Supplement 1: Notes**

**Note on deviations from protocol:**

1. In our protocol we stated our second aim would examine whether children’s pre-existing mental health problems, other pre-existing developmental disorders, family conflict and sibling conflict relate to specific trajectories. After careful consideration of previous research (Anonymous et al., in press) we found that parental psychological distress and friendship quality were significant predictors of mental health trajectories, thus we decided to include these variables in our analysis.
2. In our protocol we stated we would use continuous scores for peer-victimization and peer-aggression. However, problems with model convergence led us to create binary variables. Responses were dichotomized by “*not at all*” coded as 0 and “somewhat true” or “certainly true” coded as 1.
3. In our protocol we stated we would combine the SDQ subscale scores in “internalizing” and “externalizing”, however we decided to look at each scale (emotional, conduct and hyperactivity/inattention problems) separately to obtain a more accurate depiction of mental health difficulties.
4. In our protocol we stated we would combine three measures for a variable of intrafamilial conflict. We re-named this variable family conflict and only included two items (“*My child has arguments with siblings”* and “*My child and I argue a lot”)* as these were the only two items available. An item for partner conflict was not available.

**Note on SDQ Scoring:**

The scoring used for the SDQ bands can be found here: [https://www.sdqinfo.org/py/sdqinfo/b3.py?language=Englishqz(UK).](https://www.sdqinfo.org/py/sdqinfo/b3.py?language=Englishqz(UK)%20.) See the For Scoring section. Scores were coded based on the 4-categorisation band for the parent-rated SDQ and examined continuously (close to average = low, slightly raised = moderate, high and very high). Scores of 9 and 10 were excluded from the conduct subscale in the peer aggression model. The SDQ item in the conduct subscale “*Often fights with other children or bullies them*” was used to calculate the total score for the conduct subscale in the peer victimization model. This item was excluded from the total score for the conduct subscale in the peer aggression model (as it is used as an outcome). As a result the following SDQ bands were used to account for this changes (in bold):

Conduct subscale without peer aggression item (total score of 8): "0" = 1, "1" = 1, "2" = 1, "3" = 2, "4" = 3, "5" = 3, "6" = 4, "7" = 4, "8" = 4.

Conduct subscale with the peer aggression item (total score of 10): "0" = 1, "1" = 1, "2" = 1, "3" = 2, "4" = 3, "5" = 3, "6" = 4, "7" = 4, "8" = 4, **"9" = 4, "10" = 4.**

The total score for the emotional subscale and hyperactivity/inattention was calculated based on all items:

Emotional symptoms subscale: "0" = 1, "1" = 1, "2" = 1, "3" = 1, "4" = 2, "5" = 3, "6" = 3, "7" = 4, "8" = 4, "9" = 4, "10" = 4

Hyperactivity/Inattention subscale: "0" = 1, "1" = 1, "2" = 1, "3" = 1, "4" = 1, "5" = 1, "6" = 2, "7" = 2, "8" = 3, "9" = 4, "10" = 4)

**Supplement 2: Missing Data and Attrition**

Attrition was difficult to determine because respondents were allowed to complete surveys at any follow-up time point. Figure S1 shows that 435 parents completed two surveys including the baseline survey, most parents in our sample completed 2-4 out of 13 surveys with SDQ items, including the baseline survey. 127 parents completed all SDQ items in the survey for all time points. Figure S2 shows that over time less parents completed surveys thus there was more missing data in the later time points.

**Figure S1.**

*Non-cumulative frequency of surveys with all SDQ items completed by a parent across all time points*


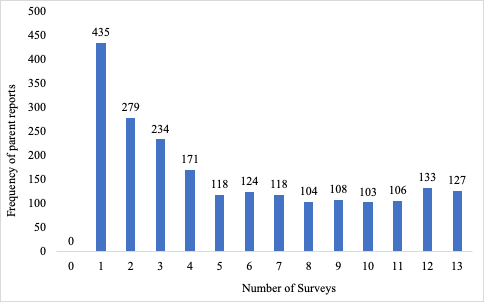


*Note*: participants had to complete a baseline survey (e.g. no missing data) and at least one other time point to

be included in the study, thus there is no missing data at baseline.

**Figure S2.**

*Non-cumulative missing data patterns across SDQ subscales for each time-point*

*Note*: Missing data was equal across SDQ subscales. This figure shows the number of surveys in our sample (n = 2,160) where the SDQ was not completed for a particular wave. No baseline (T0) data is missing per inclusion criteria.

**Supplement 3: GLM Model Specifications**

For this analysis emotional, conduct and hyperactivity/inattention subscales were aggregated into a total mental health symptoms variable. Peer victimization and peer aggression remained the same. Mental health and peer problem variables were averaged across 3 time points for T1 (pre lockdown - October, November and December), 2 time points for T2 (lockdown – January and February) and 3 time points for T3 (post lockdown – March, April and May). When only 1 score was available for any time point (e.g. January of Lockdown) these were used.

Generalized linear models (GLM) were used to examine pathways between mental health and individual peer problems. This analysis requires complete cases, therefore only participants with no missing data on these varaibles were included in the analysis (N=752). Four GLM models were used – two examining bidirectional associations between mental health and peer victimization and two examining bidirectional associations between mental health and peer aggression. Binomial distributions were used for mental health outcomes and Gaussian distributions for peer problem outcomes.

**Supplementary Table S1.**

*Generalized linear regression results for all pathways between peer victimization and mental health*

| Model N=752 | Means (SD) | B | S.E | p-value | OR | 95% CI |
| --- | --- | --- | --- | --- | --- | --- |
| peer victimization (post- lockdown) | 0.22 (0.42) |  | | | | |
| mental health T1 (pre-lockdown) | 9.43 (3.62) | 0.03 | .056 | .538 | 1.04 | 0.93 – 1.16 |
| mental health T2 (lockdown) | 10.17 (3.69) | 0.18 | .058 | .001 | 1.20 | 1.07 – 1.35 |
| peer victimization (pre-lockdown) | 0.22 (0.41) | 2.34 | .309 | <.001 | 10.41 | 5.70 – 19.23 |
| peer victimization (lockdown) | 0.23 (0.42) | 2.33 | .294 | <.001 | 10.27 | 5.78 – 18.37 |
| R^2^ = 0.597 | | | | | | |
|  | **Means (S.D)** | **B** | **S.E** | **p-value** | **t** | **95% CI** |
| mental health (post-lockdown) | 9.89 (3.66) |  |  |  |  |  |
| mental health T1 (pre-lockdown) | 9.43 (3.62) | 0.37 | .031 | <.001 | 11.82 | 0.31 – 0.43 |
| mental health T2 (lockdown) | 10.17 (3.69) | 0.51 | .030 | <.001 | 17.04 | 0.45 – 0.57 |
| peer victimization (pre-lockdown) | 0.22 (0.41) | 0.27 | .229 | .233 | 1.20 | -0.17 – 0.72 |
| peer victimization (lockdown) | 0.23 (0.42) | 0.27 | .226 | .229 | 1.20 | -0.17 – 0.72 |
| R^2^ = 0.746 | | | | | | |

Note: OR is used for binary logistic regression (binary outcomes) and t-values for linear regression (continuous outcomes).

**Table S2.**

*Generalized linear regression results for all pathways between peer aggression and mental health*

| Model N=752 | Means (S.D) | B | S.E | p-value | OR | 95% CI |
| --- | --- | --- | --- | --- | --- | --- |
| Peer aggression (post-lockdown) | 0.09 (0.28) |  | | | | |
| mental health T1 (pre-lockdown) | 9.33 (3.52) | 0.12 | .070 | .130 | 1.11 | 0.97 – 1.28 |
| mental health T2 (lockdown) | 10.07 (3.60) | 0.06 | .072 | .400 | 1.06 | 0.92 – 1.22 |
| peer aggression (pre-lockdown) | 0.10 (0.30) | 1.27 | .408 | .002 | 3.58 | 1.61 – 7.96 |
| peer aggression (lockdown) | 0.11 (0.31) | 2.78 | .401 | <.001 | 16.14 | 7.35 – 35.43 |
| R^2^ = 0.597.626 | | | | | | |
|  | **Means (S.D)** | **B** | **S.E** | **p-value** | **t** | **95% CI** |
| Mental health (post-lockdown) | 9.80 (3.57) |  |  |  |  |  |
| mental health T1 (pre-lockdown) | 9.33 (3.52) | 0.40 | .031 | <.001 | 13.00 | 0.34 – 0.46 |
| mental health T2 (lockdown) | 10.07 (3.60) | 0.51 | .030 | <.001 | 16.76 | 0.45 – 0.57 |
| peer aggression (pre-lockdown) | 0.10 (0.30) | -0.46 | .287 | .112 | -1.59 | -1.02 – 0.11 |
| peer aggression (lockdown) | 0.11 (0.31) | 0.47 | .279 | .090 | 1.70 | -0.07 – 1.02 |
| R^2^ = 0.746 | | | | | | |

Note: OR is used for binary logistic regression (binary outcomes) and t-values for linear regresion (continuous outcomes)

**Supplement 4: GBTM Model Specifications**

**Table S3.**

*Model selection results*

| Number of groups | BIC | AIC |
| --- | --- | --- |
| *Peer Victimization* |  |  |
| 1 | -51848.49 | -51811.59 |
| 2 | -46848.52 | -46780.39 |
| 3 | -45635.35 | -45535.99 |
| 4 | -44913.40 | -44782.80 |
| 5 | -44315.01 | -44150.35 |
| 6 | -43966.33 | -43773.29 |
| 7 | -43547.46 | -43323.19 |
| 8 | -43282.04 | -43026.54 |
| *Peer Aggression* |  |  |
| 1 | -48372.43 | -48335.52 |
| 2 | -43962.99 | -43894.85 |
| 3 | -42936.40 | -42837.03 |
| 4 | -42099.98 | -41963.71 |
| 5 | -41637.53 | -41475.71 |
| 6 | -41235.65 | -41042.61 |
| 7 | -40920.28 | -40696.00 |
| 8 | -40769.38 | -40513.88 |

Note: BIC – Bayesian information criterion; AIC – Akaike information criterion.

To determine the number of groups we fitted a series of models between one and eight trajectories with cubic, quadratic and linear functions. We determined that quadratic functions had the best fit in most models and a five-trajectory and a four-trajectory model had the best fit and interpretability for peer victimization and peer aggression respectively. We then improved model fit by examining individual trajectory shapes. We determined that emotional symptoms followed a quadratic trend in all groups, conduct and hyperactivity problems followed a linear trend in all groups and peer victimization and peer aggression items followed a combination of mostly quadratic and cubic trends. We also examined individual trajectory functions for N-1 and N+1 trajectory groups to make sure model fit and interpretability was still best for a N=5 or N=4 trajectory models. For the sake of comparison, this figure shows model fit indices BIC and AIC based on quadratic functions for all exposures.

**Table S4.**

*Model Adequacy Results*

| Trajectory group | N(%) | A*ve*PP | OCC |
| --- | --- | --- | --- |
| Model: Peer Victimization |  |  |  |
| Low MH no PV | 759 (34.3) | 0.92 | 20 |
| mod MH low PA | 401 (19.2) | 0.83 | 22 |
| Mod MH high VP | 211 (9.9) | 0.87 | 61 |
| Mod-High MH low VP | 416 (19.3) | 0.86 | 26 |
| High MH high VP | 378 (17.3) | 0.92 | 57 |
| Model: Peer Aggression |  |  |  |
| Low MH no PA | 836 (38.1) | 0.92 | 18 |
| Mod-high MH low PA | 511 (23.7) | 0.86 | 19 |
| Mod MH low PA | 455 (21.4) | 0.86 | 22 |
| High MH mod PA | 358 (16.8) | 0.92 | 59 |

Note. A*ve*PP - average posterior probability; OCC – odds of correct classification.

Membership probability (AvePP) greater than 0.70 and OCC greater than 5 represent a good model fit

**Supplement 5: Code Syntax**

**Stata code – Group trajectory modelling**

*****Clear space and set path**

clear all

cd "/Users/Desktop/STATA"

*****Import data**

use bullying_stata.dta

*****function to give me APP and OCC in one table**

program summary_table_procTraj

preserve

**

drop if missing(_traj_Group)

*now lets look at the average posterior probability

gen Mp = 0

foreach i of varlist _traj_ProbG* {

replace Mp = `i' if `i' > Mp

}

sort _traj_Group

*and the odds of correct classification

by _traj_Group: gen countG = _N

by _traj_Group: egen groupAPP = mean(Mp)

by _traj_Group: gen counter = _n

gen n = groupAPP/(1 - groupAPP)

gen p = countG/ _N

gen d = p/(1-p)

gen occ = n/d

*Estimated proportion for each group

scalar idx = 0

gen TotProb = 0

foreach i of varlist _traj_ProbG* {

scalar idx = idx + 1

quietly summarize `i'

replace TotProb = r(sum)/ _N if _traj_Group == idx

}

gen GROUP_APP = round(groupAPP*100,.1)

gen OCC = round(occ,.1)

gen Probab = round(p*100,.1)

gen Prob_post = round(TotProb*100,.1)

list _traj_Group countG GROUP_APP OCC Probab Prob_post if counter == 1

restore

end

*****Create a variable for time (month)**

gen month0=0

gen month1=1

gen month2=2

gen month3=3

gen month4=4

gen month5=5

gen month6=6

gen month7=7

gen month8=8

gen month9=9

gen month10=10

gen month11=11

gen month12=12

gen month13=13

*****Trajectories – example using the bullying model**

*1-TRAJ MODEL

traj, multgroups(1) var1(emo_0-emo_13) indep1(month0-month13) model1(cnorm) min1(1) max1(4) order1(2) var2(hyper_0-hyper_13) indep2(month0-month13) model2(cnorm) min2(1) max2(4) order2() var3(confights_0-confights_13) indep3(month0-month13) model3(cnorm) min3(1) max3(4) order3(1) var4(bullied_0-bullied_13) indep4(month0-month13) model4(logit) order4(2)

summary_table_procTraj

*2-TRAJ MODEL

traj, multgroups(2) var1(emo_0-emo_13) indep1(month0-month13) model1(cnorm) min1(1) max1(4) order1(2 2) var2(hyper_0-hyper_13) indep2(month0-month13) model2(cnorm) min2(1) max2(4) order2(1 1) var3(confights_0-confights_13) indep3(month0-month13) model3(cnorm) min3(1) max3(4) order3(1 1) var4(bullied_0-bullied_13) indep4(month0-month13) model4(logit) order4(2 2)

summary_table_procTraj

*3-TRAJ MODEL

traj, multgroups(3) var1(emo_0-emo_13) indep1(month0-month13) model1(cnorm) min1(1) max1(4) order1(2 2 2) var2(hyper_0-hyper_13) indep2(month0-month13) model2(cnorm) min2(1) max2(4) order2(1 1 1) var3(confights_0-confights_13) indep3(month0-month13) model3(cnorm) min3(1) max3(4) order3(1 1 1) var4(bullied_0-bullied_13) indep4(month0-month13) model4(logit) order4(2 2 2)

summary_table_procTraj

*4-TRAJ MODEL

traj, multgroups(4) var1(emo_0-emo_13) indep1(month0-month13) model1(cnorm) min1(1) max1(4) order1(2 2 2 2) var2(hyper_0-hyper_13) indep2(month0-month13) model2(cnorm) min2(1) max2(4) order2(1 1 1 1) var3(confights_0-confights_13) indep3(month0-month13) model3(cnorm) min3(1) max3(4) order3(1 1 1 1) var4(bullied_0-bullied_13) indep4(month0-month13) model4(logit) order4(2 2 2 2)

summary_table_procTraj

*5-TRAJ MODEL

traj, multgroups(5) var1(emo_0-emo_13) indep1(month0-month13) model1(cnorm) min1(1) max1(4) order1(2 2 2 2 2) var2(hyper_0-hyper_13) indep2(month0-month13) model2(cnorm) min2(1) max2(4) order2(1 1 1 1 1) var3(confights_0-confights_13) indep3(month0-month13) model3(cnorm) min3(1) max3(4) order3(1 1 1 1 1) var4(bullied_0-bullied_13) indep4(month0-month13) model4(logit) order4(2 2 2 2 3)

summary_table_procTraj

***Plot final model**

multtrajplot, xtitle("Month") ytitle1("Emotion") ytitle2("Hyperactivity/Inattention") ytitle3("Conduct") ytitle4("Peer Victimization") ylabel1(0(1)4) ylabel2(0(1)4) ylabel3(0(1)4) ylabel4(0(0.2)1) xlabel(0(2)13)

*****Trajectories – examples using the peer aggression model**

*1-TRAJ MODEL

traj, multgroups(1) var1(emo_0-emo_13) indep1(month0-month13) model1(cnorm) min1(1) max1(4) order1(2) var2(hyper_0-hyper_13) indep2(month0-month13) model2(cnorm) min2(1) max2(4) order2(1) var3(con_0-con_13) indep3(month0-month13) model3(cnorm) min3(1) max3(4) order3(1) var4(fights_0-fights_13) indep4(month0-month13) model4(logit) order4(2)

*2-TRAJ MODEL

traj, multgroups(2) var1(emo_0-emo_13) indep1(month0-month13) model1(cnorm) min1(1) max1(4) order1(2 2) var2(hyper_0-hyper_13) indep2(month0-month13) model2(cnorm) min2(1) max2(4) order2(1 1) var3(con_0-con_13) indep3(month0-month13) model3(cnorm) min3(1) max3(4) order3(1 1) var4(fights_0-fights_13) indep4(month0-month13) model4(logit) order4(2 2)

*3-TRAJ MODEL

traj, multgroups(3) var1(emo_0-emo_13) indep1(month0-month13) model1(cnorm) min1(1) max1(4) order1(2 2 2) var2(hyper_0-hyper_13) indep2(month0-month13) model2(cnorm) min2(1) max2(4) order2(1 1 1) var3(con_0-con_13) indep3(month0-month13) model3(cnorm) min3(1) max3(4) order3(1 1 1) var4(fights_0-fights_13) indep4(month0-month13) model4(logit) order4(2 2 2)

*4-TRAJ MODEL

traj, multgroups(4) var1(emo_0-emo_13) indep1(month0-month13) model1(cnorm) min1(1) max1(4) order1(2 2 2 2) var2(hyper_0-hyper_13) indep2(month0-month13) model2(cnorm) min2(1) max2(4) order2(1 1 1 1) var3(con_0-con_13) indep3(month0-month13) model3(cnorm) min3(1) max3(4) order3(1 1 1 1) var4(fights_0-fights_13) indep4(month0-month13) model4(logit) order4(2 2 3 3)

*****Plot final model**

multtrajplot, xtitle("Month") ytitle1("Emotion") ytitle2("Hyper") ytitle3("Conduct") ytitle4("Fights") ylabel1(0(1)4) ylabel2(0(1)4) ylabel3(0(1)4) ylabel4(0(0.2)0.8) xlabel(0(2)13)

**Supplement 6: Multinomial Regression Analysis**

**Table S5.** *Sensitivity analysis for family conflict variable*

| **Predictor** | **Odds Ratios 95% CI** | **P value** |
| --- | --- | --- |
| **Peer Victimization** | | |
| **Low Reference** |  |  |
| family conflict 2 * mod MH/low PV | 1.75 (1.52 – 2.00) | **<.001** |
| Family conflict 2 * mod MH/high PV | 1.37 (1.16 – 1.63) | **<.001** |
| family conflict 2 * mod-high MH/low PV | 1.39 (1.21 – 1.59) | **<.001** |
| family conflict 2 * high MH/high PV | 2.13 (1.84 – 2.48) | **<.001** |
| **High Reference** |  |  |
| family conflict 2 * low MH/no PV | 0.47 (0.40 – 0.54) | **<.001** |
| family conflict 2 * mod MH/low PV | 0.82 (0.71 – 0.94) | **.006** |
| Family conflict 2 * mod MH/high PV | 0.64 (0.54 – 0.77) | **<.001** |
| family conflict 2 * mod-high MH/low PV | 0.65 (0.56 – 0.75) | **<.001** |
| **Peer Aggression** | | |
| **Low Reference** |  |  |
| family conflict 2 * mod-high MH/low PA | 1.27 (1.12 – 1.45) | **<.001** |
| family conflict 2 * mod MH/low PA | 1.68 (1.48 – 1.91) | **<.001** |
| family conflict 2 * high MH/mod PA | 2.10 (1.82 – 2.43) | **<.001** |
| **High Reference** |  |  |
| family conflict 2 * low MH/no PA | 0.48 (0.41 – 0.55) | **<.001** |
| family conflict 2 * mod-high MH/low PA | 0.61 (0.53 – 0.70) | **<.001** |
| family conflict 2 * mod MH/low PA | 0.80 (0.70 – 0.91) | **.001** |

Note: the family conflict variable in this analysis includes scores from children with and without siblings. When compared to the family conflict variable in the main analysis (that only includes scores from children with sibling) the results are the same.

**Table S6.** *MNRA peer victimization – Low Stable Reference*

| **Predictors** | **Odds Ratios 95% CI** | **P value** |
| --- | --- | --- |
| (Intercept) * MOD MH/LOW PV | 0.40 (0.10 – 1.63) | .199 |
| (Intercept) * MOD MH/HIGH PV | 0.68 (0.15 – 3.20) | .628 |
| (Intercept) * MOD-HIGH MH/LOW PV | 0.69 (0.18 – 2.70) | .596 |
| (Intercept) * HIGH MH/HIGH PV | 0.13 (0.03 – 0.59) | .**008** |
| child gender [M] * MOD MH/LOW PV | 1.26 (0.92 – 1.72) | .146 |
| child gender [M] * MOD MH/HIGH PV | 1.36 (0.90 – 2.06) | .147 |
| child gender [M] * MOD-HIGH MH/LOW PV | 0.76 (0.56 – 1.04) | .085 |
| child gender [M] * HIGH MH/HIGH PV | 0.97 (0.67 – 1.41) | .893 |
| child ethnicity [White] * MOD MH/LOW PV | 0.81 (0.46 – 1.45) | .480 |
| child ethnicity [White] * MOD MH/HIGH PV | 1.10 (0.49 – 2.47) | .816 |
| child ethnicity [White] * MOD-HIGH MH/LOW PV | 1.08 (0.58 – 2.01) | .799 |
| child ethnicity [White] * HIGH MH/HIGH PV | 1.38 (0.65 – 2.93) | .398 |
| income2 [>£16k] * MOD MH/LOW PV | 0.29 (0.08 – 1.02) | .053 |
| income2 [>£16k] * MOD MH/HIGH PV | 0.12 (0.03 – 0.43) | **.001** |
| income2 [>£16k] * MOD-HIGH MH/LOW PV | 0.21 (0.07 – 0.70) | **.011** |
| income2 [>£16k] * HIGH MH/HIGH PV | 0.12 (0.04 – 0.42) | **.001** |
| SENND [Yes] * MOD MH/LOW PV | 2.98 (1.48 – 6.01) | **.002** |
| SENND [Yes] * MOD MH/HIGH PV | 7.61 (3.74 – 15.49) | **<.001** |
| SENND [Yes] * MOD-HIGH MH/LOW PV | 2.48 (1.19 – 5.18) | **.016** |
| SENND [Yes] * HIGH MH/HIGH PV | 14.02 (7.23 – 27.19) | **<.001** |
| dass 9item * MOD MH/LOW PV | 1.10 (1.05 – 1.15) | **<.001** |
| dass 9item * MOD MH/HIGH PV | 1.05 (0.99 – 1.12) | .084 |
| dass 9item * MOD-HIGH MH/LOW PV | 1.17 (1.13 – 1.22) | **<.001** |
| dass 9item * HIGH MH/HIGH PV | 1.26 (1.20 – 1.31) | **<.001** |
| family conflict * MOD MH/LOW PV | 2.02 (1.69 – 2.41) | **<.001** |
| family conflict * MOD MH/HIGH PV | 1.63 (1.30 – 2.04) | **<.001** |
| family conflict * MOD-HIGH MH/LOW PV | 1.54 (1.29 – 1.84) | **<.001** |
| family conflict * HIGH MH/HIGH PV | 2.64 (2.17 – 3.21) | **<.001** |
| One friend [Yes] * MOD MH/LOW PV | 0.60 (0.44 – 0.82) | **.001** |
| One friend [Yes] * MOD MH/HIGH PV | 0.33 (0.22 – 0.50) | **<.001** |
| One friend [Yes] * MOD-HIGH MH/LOW PV | 0.58 (0.43 – 0.80) | **.001** |
| One friend [Yes] * HIGH MH/HIGH PV | 0.23 (0.15 – 0.34) | **<.001** |

**Table S7.** *MNRA peer victimization – High Stable Reference*

| **Predictors** | **Odds Ratios 95% CI** | **P value** |
| --- | --- | --- |
| (Intercept) * LOW MH/NO PV | 7.57 (1.70 – 33.81) | **.008** |
| (Intercept) * MOD MH/LOW PV | 3.01 (0.82 – 10.96) | .095 |
| (Intercept) * MOD MH/HIGH PV | 5.16 (1.24 – 21.58) | **.024** |
| (Intercept) * MOD-HIGH MH/LOW PV | 5.24 (1.50 – 18.28) | **.009** |
| child gender [M] * LOW MH/NO PV | 1.03 (0.71 – 1.49) | .893 |
| child gender [M] * MOD MH/LOW PV | 1.29 (0.88 – 1.89) | .191 |
| child gender [M] * MOD MH/HIGH PV | 1.39 (0.88 – 2.22) | .161 |
| child gender [M] * MOD-HIGH MH/LOW PV | 0.78 (0.54 – 1.14) | .205 |
| child ethnicity [White] * LOW MH/NO PV | 0.72 (0.34 – 1.53) | .398 |
| child ethnicity [White] * MOD MH/LOW PV | 0.59 (0.28 – 1.22) | .152 |
| child ethnicity [White] * MOD MH/HIGH PV | 0.80 (0.32 – 1.98) | .624 |
| child ethnicity [White] * MOD-HIGH MH/LOW PV | 0.78 (0.37 – 1.68) | .531 |
| income [>£16k] * LOW MH/NO PV | 8.15 (2.40 – 27.70) | **.001** |
| income [>£16k] * MOD MH/LOW PV | 2.37 (0.91 – 6.18) | .077 |
| income [>£16k] * MOD MH/HIGH PV | 1.00 (0.39 – 2.57) | .998 |
| income [>£16k] * MOD-HIGH MH/LOW PV | 1.74 (0.73 – 4.13) | .210 |
| SENND [Yes] * LOW MH/NO PV | 0.07 (0.04 – 0.14) | **<.001** |
| SENND [Yes] * MOD MH/LOW PV | 0.21 (0.13 – 0.35) | **<.001** |
| SENND [Yes] * MOD MH/HIGH PV | 0.54 (0.32 – 0.93) | **.0250** |
| SENND [Yes] * MOD-HIGH MH/LOW PV | 0.18 (0.10 – 0.31) | **<.001** |
| dass 9item * LOW MH/NO PV | 0.80 (0.76 – 0.83) | **<.001** |
| dass 9item * MOD MH/LOW PV | 0.88 (0.84 – 0.91) | **<.001** |
| dass 9item * MOD MH/HIGH PV | 0.8(0.79 – 0.89) | **<.001** |
| dass 9item * MOD-HIGH MH/LOW PV | 0.93 (0.90 – 0.97) | **.001** |
| family conflict * LOW MH/NO PV | 0.38 (0.31 – 0.46) | **<.001** |
| family conflict * MOD MH/LOW PV | 0.76 (0.64 – 0.91) | **.003** |
| family conflict * MOD MH/HIGH PV | 0.62 (0.49 – 0.77) | **<.001** |
| family conflict * MOD-HIGH MH/LOW PV | 0.58 (0.48 – 0.70) | **<.001** |
| One friend [Yes] * LOW MH/NO PV | 4.36 (2.93 – 6.47) | **<.001** |
| One friend [Yes] * MOD MH/LOW PV | 2.62 (1.74 – 3.93) | **<.001** |
| One friend [Yes] * MOD MH/HIGH PV | 1.44 (0.87 – 2.36) | .154 |
| One friend [Yes] * MOD-HIGH MH/LOW PV | 2.55 (1.70 – 3.82) | **<.001** |

**Table S8.** *MNRA peer victimization – Mod MH/ High PV*

| **Predictors** | **Odds Ratios 95% CI** | **P value** |
| --- | --- | --- |
| (Intercept) * LOW MH/NO PV | 1.52 (0.32 – 7.15) | .597 |
| (Intercept) * MOD MH/LOW PV | 0.58 (0.14 – 2.44) | .458 |
| (Intercept) * MOD-HIGH MH/LOW PV | 0.96 (0.24 – 3.92) | .959 |
| (Intercept) * HIGH MH/HIGH PV | 0.18 (0.04 – 0.76) | **.020** |
| child gender [M] * LOW MH/NO PV | 0.73 (0.48 – 1.11) | .143 |
| child gender [M] * MOD MH/LOW PV | 0.92 (0.59 – 1.43) | .716 |
| child gender [M] * MOD-HIGH MH/LOW PV | 0.56 (0.36 – 0.86) | **.009** |
| child gender [M] * HIGH MH/HIGH PV | 0.71 (0.44 – 1.13) | .144 |
| child ethnicity [White] * LOW MH/NO PV | 0.90 (0.40 – 2.02) | .796 |
| child ethnicity [White] * MOD MH/LOW PV | 0.74 (0.32 – 1.68) | .468 |
| child ethnicity [White] * MOD-HIGH MH/LOW PV | 1.01 (0.43 – 2.38) | .990 |
| child ethnicity [White] * HIGH MH/HIGH PV | 1.28 (0.52 – 3.19) | .591 |
| child MH [Yes] * LOW MH/NO PV | 0.00 (0.00 – Inf) | .996 |
| child MH [Yes] * MOD MH/LOW PV | 0.35 (0.03 – 3.86) | .394 |
| child MH [Yes] * MOD-HIGH MH/LOW PV | 2.97 (0.58 – 15.27) | .193 |
| child MH [Yes] * HIGH MH/HIGH PV | 4.67 (1.03 – 21.19) | **.046** |
| income [>£16k] * LOW MH/NO PV | 8.02 (2.25 – 28.60) | **.001** |
| income [>£16k] * MOD MH/LOW PV | 2.38 (0.82 – 6.96) | .112 |
| income [>£16k] * MOD-HIGH MH/LOW PV | 1.78 (0.65 – 4.84) | .260 |
| income [>£16k] * HIGH MH/HIGH PV | 1.03 (0.40 – 2.69) | .948 |
| SENND [Yes] * LOW MH/NO PV | 0.14 (0.07 – 0.29) | **<.001** |
| SENND [Yes] * MOD MH/LOW PV | 0.40 (0.22 – 0.74) | **.003** |
| SENND [Yes] * MOD-HIGH MH/LOW PV | 0.30 (0.15 – 0.58) | **<.001** |
| SENND [Yes] * HIGH MH/HIGH PV | 1.60 (0.93 – 2.76) | .091 |
| dass 9item * LOW MH/NO PV | 0.95 (0.89 – 1.01) | .082 |
| dass 9item * MOD MH/LOW PV | 1.04 (0.98 – 1.11) | .158 |
| dass 9item * MOD-HIGH MH/LOW PV | 1.11 (1.05 – 1.18) | **<.001** |
| dass 9item * HIGH MH/HIGH PV | 1.19 (1.12 – 1.26) | **<.001** |
| family conflict * LOW MH/NO PV | 0.62 (0.49 – 0.77) | **<.001** |
| family conflict * MOD MH/LOW PV | 1.25 (0.99 – 1.56) | .055 |
| family conflict * MOD-HIGH MH/LOW PV | 0.95 (0.75 – 1.20) | .658 |
| family conflict * HIGH MH/HIGH PV | 1.62 (1.29 – 2.04) | **<.001** |
| One friend [Yes] * LOW MH/NO PV | 3.02 (1.98 – 4.60) | **.001** |
| One friend [Yes] * MOD MH/LOW PV | 1.82 (1.17 – 2.85) | **.008** |
| One friend [Yes] * MOD-HIGH MH/LOW PV | 1.77 (1.13 – 2.77) | **.013** |
| One friend [Yes] * HIGH MH/HIGH PV | 0.68 (0.42 – 1.13) | .137 |

**Table S9.** *MNRA peer victimization – Mod-High MH/ Low PV*

| **Predictors** | **Odds Ratios 95% CI** | **P value** |
| --- | --- | --- |
| (Intercept) * LOW MH/NO PV | 1.58 (0.40 – 6.24) | .518 |
| (Intercept) * MOD MH/LOW PV | 0.60 (0.17 – 2.09) | .426 |
| (Intercept) * MOD MH/HIGH PV | 1.04 (0.26 – 4.21) | .959 |
| (Intercept) * HIGH MH/HIGH PV | 0.19 (0.05 – 0.66) | **.009** |
| child gender [M] * LOW MH/NO PV | 1.32 (0.97 – 1.79) | .081 |
| child gender [M] * MOD MH/LOW PV | 1.65 (1.17 – 2.33) | **.004** |
| child gender [M] * MOD MH/HIGH PV | 1.79 (1.16 – 2.79) | **.009** |
| child gender [M] * HIGH MH/HIGH PV | 1.27 (0.87 – 1.86) | .223 |
| child ethnicity [White] * LOW MH/NO PV | 0.89 (0.48 – 1.67) | .724 |
| child ethnicity [White] * MOD MH/LOW PV | 0.73 (0.38 – 1.40) | .347 |
| child ethnicity [White] * MOD MH/HIGH PV | 0.99 (0.42 – 2.35) | .990 |
| child ethnicity [White] * HIGH MH/HIGH PV | 1.28 (0.60 – 2.74) | .530 |
| child MH [Yes] * LOW MH/NO PV | 0.00 (0.00 – Inf) | .995 |
| child MH [Yes] * MOD MH/LOW PV | 0.12 (0.01 – 0.96) | **.045** |
| child MH [Yes] * MOD MH/HIGH PV | 0.34 (0.07 – 1.73) | .193 |
| child MH [Yes] * HIGH MH/HIGH PV | 1.57 (0.57 – 4.37) | .386 |
| Income [>£16k] * LOW MH/NO PV | 4.51 (1.36 – 14.95) | **.014** |
| income [>£16k] * MOD MH/LOW PV | 1.34 (0.50 – 3.59) | .559 |
| income [>£16k] * MOD MH/HIGH PV | 0.56 (0.21 – 1.53) | .260 |
| income [>£16k] * HIGH MH/HIGH PV | 0.58 (0.24 – 1.39) | .222 |
| SENND [Yes] * LOW MH/NO PV | 0.47 (0.22 – 1.00) | **.050** |
| SENND [Yes] * MOD MH/LOW PV | 1.35 (0.71 – 2.57) | .364 |
| SENND [Yes] * MOD MH/HIGH PV | 3.35 (1.73 – 6.49) | **<.001** |
| SENND [Yes] * HIGH MH/HIGH PV | 5.36 (3.01 – 9.52) | **<.001** |
| dass 9item * LOW MH/NO PV | 0.85 (0.82 – 0.89) | **<.001** |
| dass 9item * MOD MH/LOW PV | 0.94 (0.90 – 0.98) | **.002** |
| dass 9item * MOD MH/HIGH PV | 0.90 (0.85 – 0.95) | **<.001** |
| dass 9item * HIGH MH/HIGH PV | 1.07 (1.03 – 1.11) | **<.001** |
| family conflict * LOW MH/NO PV | 0.65 (0.54 – 0.78) | **<.001** |
| family conflict * MOD MH/LOW PV | 1.31 (1.10 – 1.58) | **.003** |
| family conflict * MOD MH/HIGH PV | 1.05 (0.84 – 1.33) | .658 |
| family conflict * HIGH MH/HIGH PV | 1.71 (1.42 – 2.06) | **<.001** |
| one friend [Yes] * LOW MH/NO PV | 1.71 (1.25 – 2.33) | **.001** |
| one friend [Yes] * MOD MH/LOW PV | 1.03 (0.73 – 1.45) | .866 |
| one friend [Yes] * MOD MH/HIGH PV | 0.57 (0.36 – 0.88) | **.013** |
| one friend [Yes] * HIGH MH/HIGH PV | 0.39 (0.26 – 0.58) | **<.001** |

**Table S10.** *MNRA peer aggression – Low Stable Reference*

| **Predictors** | **Odds Ratios 95% CI** | **P value** |
| --- | --- | --- |
| (Intercept) * MOD-HIGH MH/LOW PA | 0.79 (0.23 – 2.70) | .706 |
| (Intercept) * MOD MH/LOW PA | 0.31 (0.08 – 1.13) | .076 |
| (Intercept) * HIGH MH/MOD PA | 0.11 (0.03 – 0.46) | **.002** |
| child gender [M] * MOD-HIGH MH/LOW PA | 0.87 (0.66 – 1.16) | .340 |
| child gender [M] * MOD MH/LOW PA | 1.40 (1.03 – 1.90) | **.032** |
| child gender [M] * HIGH MH/MOD PA | 0.63 (0.44 – 0.91) | **.015** |
| child ethnicity [White] * MOD-HIGH MH/LOW PA | 1.36 (0.76 – 2.41) | .302 |
| child ethnicity [White] * MOD MH/LOW PA | 0.95 (0.54 – 1.66) | .845 |
| child ethnicity [White] * HIGH MH/MOD PA | 1.64 (0.78 – 3.42) | .190 |
| income [>£16k] * MOD-HIGH MH/LOW PA | 0.22 (0.07 – 0.62) | **.005** |
| income [>£16k] * MOD MH/LOW PA | 0.27 (0.09 – 0.84) | **.024** |
| income [>£16k] * HIGH MH/MOD PA | 0.12 (0.04 – 0.39) | **<.001** |
| SENND [Yes] * MOD-HIGH MH/LOW PA | 1.76 (0.97 – 3.19) | .063 |
| SENND [Yes] * MOD MH/LOW PA | 3.05 (1.75 – 5.31) | **<.001** |
| SENND [Yes] * HIGH MH/MOD PA | 7.87 (4.46 – 13.88) | **<.001** |
| dass 9item * MOD-HIGH MH/LOW PA | 1.16 (1.12 – 1.21) | **<.001** |
| dass 9item * MOD MH/LOW PA | 1.11 (1.07 – 1.16) | **<.001** |
| dass 9item * HIGH MH/MOD PA | 1.26 (1.20 – 1.31) | **<.001** |
| family conflict * MOD-HIGH MH/LOW PA | 1.36 (1.15 – 1.60) | **<.001** |
| family conflict * MOD MH/LOW PA | 2.05 (1.73 – 2.43) | **<.001** |
| family conflict * HIGH MH/MOD PA | 2.58 (2.13 – 3.12) | **<.001** |
| one friend [Yes] * MOD-HIGH MH/LOW PA | 0.59 (0.44 – 0.78) | **<.001** |
| one friend [Yes] * MOD MH/LOW PA | 0.53 (0.39 – 0.71) | **<.001** |
| one friend [Yes] * HIGH MH/MOD PA | 0.33 (0.23 – 0.49) | **<.001** |

**Table S11.** *MNRA peer aggression – High Stable Reference*

| **Predictors** | **Odds Ratio 95% CI** | **P value** |
| --- | --- | --- |
| (Intercept) * LOW MH/NO PA | 8.92 (2.17 – 36.71) | **.002** |
| (Intercept) * MOD-HIGH MH/LOW PA | 7.04 (2.15 – 23.09) | **.001** |
| (Intercept) * MOD MH/LOW PA | 2.74 (0.83 – 9.01) | .097 |
| child gender [M] * LOW MH/NO PA | 1.58 (1.09 – 2.28) | **.015** |
| child gender [M] * MOD-HIGH MH/LOW PA | 1.38 (0.95 – 1.99) | .090 |
| child gender [M] * MOD MH/LOW PA | 2.20 (1.51 – 3.21) | **<.001** |
| child ethnicity [White] * LOW MH/NO PA | 0.61 (0.29 – 1.28) | .190 |
| child ethnicity [White] * MOD-HIGH MH/LOW PA | 0.83 (0.39 – 1.76) | .624 |
| child ethnicity [White] * MOD MH/LOW PA | 0.58 (0.28 – 1.18) | .131 |
| income [>£16k] * LOW MH/NO PA | 8.05 (2.57 – 25.15) | **<.001** |
| income [>£16k] * MOD-HIGH MH/LOW PA | 1.74 (0.78 – 3.86) | .176 |
| income [>£16k] * MOD MH/LOW PA | 2.18 (0.94 – 5.04) | .069 |
| SENND [Yes] * LOW MH/NO PA | 0.13 (0.07 – 0.22) | **<.001** |
| SENND [Yes] * MOD-HIGH MH/LOW PA | 0.22 (0.13 – 0.37) | **<.001** |
| SENND [Yes] * MOD MH/LOW PA | 0.39 (0.25 – 0.61) | **<.001** |
| dass 9item * LOW MH/NO PA | 0.80 (0.76 – 0.83) | **<.001** |
| dass 9item * MOD-HIGH MH/LOW PA | 0.92 (0.89 – 0.96) | **<.001** |
| dass 9item * MOD MH/LOW PA | 0.88 (0.85 – 0.92) | **<.001** |
| family conflict * LOW MH/NO PA | 0.39 (0.32 – 0.47) | **<.001** |
| family conflict * MOD-HIGH MH/LOW PA | 0.53 (0.44 – 0.63) | **<.001** |
| family conflict * MOD MH/LOW PA | 0.79 (0.67 – 0.94) | **.007** |
| One friend [Yes] * LOW MH/NO PA | 3.00 (2.05 – 4.39) | **<.001** |
| One friend [Yes] * MOD-HIGH MH/LOW PA | 1.77 (1.20 – 2.60) | **.004** |
| One friend [Yes] * MOD MH/LOW PA | 1.58 (1.07 – 2.34) | **.022** |

----------------------------------------------------END OF SUPPLEMENT----------------------------------------------------
